# P01-010 – Anti-TNF agents in intractable FMF: four cases

**DOI:** 10.1186/1546-0096-11-S1-A14

**Published:** 2013-11-08

**Authors:** L Kaly, D Rimar, G Slobodin, N Jiries, I Rosner, M Rozenbaum

**Affiliations:** 1 rheumatology, Bnai Zion medical Center-Haifa Israel, Haifa, Israel

## Introduction

Familial Mediterranean Fever (FMF) is an autoinflammatory disease characterized by recurrent attacks of fever and serositis. A relation between FMF and Ankylosing Spondylitis (AS) has been suggested in small cohort studies, although there is no consensus regarding the role of HLA B27. Colchicine, the mainstay treatment in FMF, does not improve the axial or peripheral symptoms due to spondylarthropathy. There are controversial data about the efficacy of Tumor Necrosis Factor Alpha (TNF α) blockade in FMF patients [1].

## Case Report

We report our experience in 4 patients with intractable FMF treated with oral colchicine and supplemental weekly IV colchicine [2], that were treated with TNF α blockade for symptomatic axial spondylarthropathy.

One 26 years old man with MEFV mutations V726A and E148Q, negative for HLAB27, with concomitant ulcerative colitis was treated with infliximab and then with adalimumab; and 3 women (42, 48 and 55 years old), two of them treated with Infliximab and one treated with adalimumab. Two of the women were homozygous for the M694V mutation. All developed severe to moderate adverse events: exacerbation of FMF in 2 of them, myositis and ulcerative colitis exacerbation in the male patient, and staphylococcus aureus sepsis in another patient. Three of them had to stop the TNF α blockade treatment. One patient developed psoriatic rash, with no need to stop the treatment.

## Discussion

In our limited experience, TNF α blockade in patients with both intractable FMF and AS is not very effective and may be associated with severe adverse events. Little is known about the possible interaction between intravenous colchicine and anti-TNF treatment.

## Disclosure of interest

None declared
